# The Utility of 4D-CT Imaging in Primary Hyperparathyroidism Management in a Low-Volume Center

**DOI:** 10.3390/medicina59081415

**Published:** 2023-08-03

**Authors:** Marko Murruste, Martin Kivilo, Karri Kase, Ülle Kirsimägi, Annika Tähepõld, Kaia Tammiksaar

**Affiliations:** 1 Surgery Clinic of Tartu University Hospital, 50406 Tartu, Estonia; karri.kase@kliinikkum.ee (K.K.); ylle.kirsimagi@kliinikum.ee (Ü.K.); 2 Faculty of Medicine, University of Tartu, 50406 Tartu, Estonia; martinkivilo@gmail.com; 3 Radiology Clinic of Tartu University Hospital, 50406 Tartu, Estonia; annika.tahepold@kliinikum.ee; 4 Internal Medicine Clinic of Tartu University Hospital, 50406 Tartu, Estonia; kaia.tammiksaar@kliinikum.ee

**Keywords:** primary hyperparathyroidism, parathyroid imaging, parathyroidectomy, four-dimensional computed tomography

## Abstract

*Background:* Ultrasonography (US) and the 99mTc-sestamibi parathyroid scan (SPS) may have suboptimal accuracy when detecting the localization of enlarged parathyroid gland(s) (PTG). Therefore, the more accurate four-dimensional computed tomography scan (4D-CT) has been employed for PTG imaging. Currently, there is a paucity of data evaluating the utility of 4D-CT in low caseload settings. *Aim and Objectives:* To evaluate the impact of PTG imaging, using 4D-CT in conjunction with its intraoperatively displayed results, on the outcomes of surgical PTX. *Materials and Methods:* A single-center retrospective analysis of surgically treated patients with pHPT from 01/2010 to 01/2021 was conducted. An evaluation of the impact of the preoperative imaging modalities on the results of surgical treatment was carried out. *Results:* During the study period, 290 PTX were performed; 45 cases were excluded due to surgery for secondary, tertiary or recurrent HPT, or due to the use of alternative imaging techniques. The remaining 245 patients were included in the study. US was carried out for PTG imaging in 236 (96.3%), SPS in 93 (38.0%), and 4D-CT in 52 patients (21.2%). The use of 4D-CT was associated with a significantly higher rate of successful localization of enlarged PTG (49 cases, 94.2%) compared to US and SPS (74 cases, 31.4%, and 54 cases, 58.1%, respectively). We distinguished between three groups of patients based on preoperative imaging: (1) PTG lateralization via US or SPS in 106 (43.3%) cases; (2) precise localization of PTG via 4D-CT in 49 (20.0%) patients; and (3) in 90 cases (36.7%), PTG imaging failed to localize an enlarged gland. The group of 4D-CT localization had significantly shorter operative time, lower rate of simultaneous thyroid resections, as well as lower rate of removal of ≥2 PTG, compared to the other groups. The 4D-CT imaging was also associated with the lowest perioperative morbidity and with the lowest median PTH in the one month follow-up; however, compared to the other groups, these differences were statistically not significant. The implementation of 4D-CT (since 01/2018) was associated with a decrease in the need for redo surgery (from 11.5% to 7.3%) and significantly increased the annual case load of PTX at our institution (from 15.3 to 41.0) compared to the period before 4D-CT diagnostics. *Conclusions:* 4D-CT imaging enabled to precisely locate almost 95% of enlarged PTG in patients with pHPT. Accurate localization and intraoperatively displayed imaging results are useful guides for surgeons to make PTX a faster and safer procedure in a low-volume center.

## 1. Introduction

Primary hyperparathyroidism (pHPT) is an increasingly common endocrine disorder, reaching a prevalence of 0.3% in the general European population [1,2]. Furthermore, in the Scandinavian countries, the prevalence of pHPT has reached 2–5% in peri- and postmenopausal women [3]. The wider availability of blood calcium screening tests and ultrasonography (US) has probably led to this increase in the recent decades [4]. As surgical treatment is the only definitive cure for pHPT; a parallel increase in the prevalence of parathyroidectomies (PTX) has been seen in the past decades [5,6].

When compared to conservative management, PTX is a relatively safe and cost-effective procedure [7]. Cure rates of surgical PTX exceed 95% at centers with high expertise [5]. Although this procedure is associated with a relatively low risk of complications, the ultimate goal of surgery—normal function of the remaining parathyroid glands (PTG) manifesting as persistent normocalcemia—is not always met. This is often due to difficulties in the preoperative and intraoperative localization of pathologic PTG, leading to the persistence of pHPT and the need for redo surgery [8]. Although the need for preoperative PTG imaging has been a subject of numerous debates, it has been demonstrated that the preoperative localization of enlarged PTG facilitates the intraoperative exploration of the culprit gland. Furthermore, PTG imaging also has the potential to prevent a large number of bilateral neck explorations (BNE) and promote unilateral neck exploration or minimally invasive PTX instead [9]. Therefore, the current guidelines recommend the preoperative localization of hyperfunctioning PTG for the selection of suspect PTX candidates, as it allows for a more focused approach, reduces operation time and complications, and results in a better cosmetic outcome and greater patient satisfaction [10,11]. Although the preferred sequence of imaging continues to evolve and there exists no universally agreed algorithm, US is usually the first line modality, followed in some institutions by ^99m^Tc sestamibi parathyroid scan with subtraction imaging (SPS). However, both tests may have suboptimal localizing accuracy with a considerable rate of failed localizations. Therefore, a more accurate four-dimensional computed tomography scan (4D-CT) has been suggested for PTG imaging [12].

Another aspect affecting successful PTX and biochemical cure is surgeons’ experience. Multiple studies have reported better outcomes of parathyroid surgery in high-volume centers, however, no definite volume threshold has been established [13]. Although some studies have proposed a threshold of 20 PTX per year, this number is purely based on professional opinions, a so-called ‘pragmatic and achievable target’ [14,15].

The above-described problems were also seen in our center. We had a relatively high percentage of patients with failed preoperative localization of PTG, and our annual case-load was relatively low (ca 10–15 cases of surgically treated patients with pHPT) [16]. Thus, in 2018, we attempted to improve the PTX success rate by implementing 4D-CT imaging for the localization of pathologically enlarged PTG from 2018.

The aim of the present study was to evaluate the impact of using 4D-CT as the PTG imaging modality on the outcomes of surgical PTX.

Our primary outcome was sensitivity of 4D-CT in comparison to US and SPS for preoperative PTG adenoma localization. The secondary outcomes were the need for redo surgery and morbidity. Additionally, the characteristics of surgical PTX, i.e., operative time, number of removed parathyroid glands, and rate of simultaneous thyroid surgery, were assessed.

We hypothesized that the success rate of 4D-CT in detecting enlarged PTG would be higher compared to US and SPS, which would result in better outcomes of surgical treatment. Furthermore, we hoped that the implementation of 4D-CT would make PTX outcomes comparable to those of large volume centers.

## 2. Methods

Ethics. The present study is a single-center retrospective analysis of surgically treated patients with pHPT. The Research Ethics Committee of the University of Tartu approved this clinical research (protocol 300/T-4).

Patients. All adult patients aged 18 years or older, who were operated due to pHPT at the Department of Surgery of Tartu University Hospital between 01/2010 and 01/2021, and on whom US, SPS, or 4D-CT for PTG imaging was used, were included in the study. The exclusion criteria were surgery for secondary, tertiary, or recurrent HPT. We also excluded a few patients whose PTG imaging investigations were magnetic resonance imaging (MRI) or conventional computed tomography (CT).

**Figure 1:**
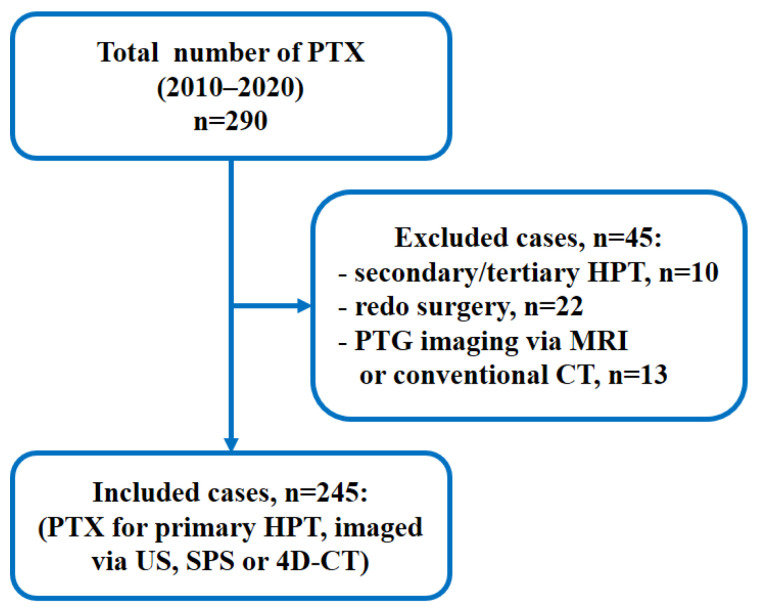
Study flowchart. PTX parathyroidectomy; HPT hyperparathyroidism; PTG parathyroid gland; US ultrasonography; SPS sestamibi parathyroid scan; 4D-CT four-dimensional computed tomography; MRI magnetic resonance imaging.

During the study period, 290 parathyroid operations were carried out; 32 cases were excluded due to surgery for secondary, tertiary, or recurrent HPT and 13 cases due to PTG imaging via MRI or conventional CT. The remaining 245 patients, who were operated for pHPT and whose imaging investigations were US, SPS, or 4D-CT, were included in the study (Figure 1).

PTG imaging. In the present study, the routine management of patients with suspected pHPT consisted of preoperative biochemical confirmation of pHPT, followed by localization investigations, including US (performed by endocrinologists) and SPS in cases of inconclusive US imaging. Since 2018, we have replaced SPS with 4D-CT imaging, and SPS was only used in cases where 4D-CT was contraindicated (e.g., renal insufficiency, intolerance of iodine based contrast media). The 4D-CT scanning protocol consists of precontrast imaging followed by arterial (25 to 30 s after contrast injection), early-delayed (40 to 45 s after contrast injection), and late-delayed (80 to 90 s after contrast injection) phases.

To facilitate better intraoperative localization of enlarged PTG, the results of 4D-CT imaging (precise localization of suspected pathological PTG) were always displayed intraoperatively using the axial, coronal, and/or sagittal CT-planes.

Surgical treatment consisted of a small Kocher incision (usually up to 5 cm) for access, followed by the classical medial approach to explore the thyroid and parathyroid regions, mobilizing the strap muscles from their midline position by progressive lateral mobilization.

For patients with failed preoperative localization or with multiglandular PTG involvement, a classical BNE was carried out. In the case of successful lateralization of enlarged PTG using US or SPS, a unilateral neck exploration was employed. For patients with precise preoperative localization of pathologic PTG via 4D-CT imaging, a one-region dissection in the suspected area was undertaken. In the case of failed focused exploration of PTG, conversion to classical BNE was employed.

Database. The study database comprises data about surgical treatment (operative time, number of removed PTG, simultaneous thyroid surgery, complications, and length of stay) and biochemical characteristics before and after surgical treatment (serum calcium, ionized calcium, parathyroid hormone (PTH), 25-hydroxyvitamin D, phosphate, and creatinine levels). Biochemical follow-up data were obtained one month and one year after surgical treatment.

Persistence of pHPT was defined as the presence of one of the following: reoperation for pHPT during 2 years of follow-up, or ionized calcium levels above 1.32 mmol/L with synchronous PTH levels over 6.9 pmol/L.

Data analysis. The impact of the preoperative imaging modalities on the results of surgical treatment were evaluated in three groups: (1) PTG localization via US or SPS, as these modalities enabled to detect the affected side (‘lateralization’), however, precise three-dimensional localization was not employed; (2) precise localization of PTG via 4D-CT; and (3) cases where preoperative PTG localization was not successful (‘image negative patients’).

An additional assessment of the results was carried out in two time frames: before the implementation of 4D-CT (from 01/2010 to 12/2017) and after it (01/2018 to 01/2021).

Statistical analysis. All perioperative and follow-up data were entered into a computerized database (Microsoft Access 2016). The software package Statistica version 13.3 (TIBCO Software, Palo Alto, CA, USA) was used for statistical calculations.

The main characteristics are presented as medians (with 25% and 75% percentiles) in the case of non-normal distribution of variables; dichotomous variables are reported as counts and percentages. Comparisons between the study groups were made using the following tests: Fisher’s exact test in the case of percentages, non-parametric Mann–Whitney U-test in the case of medians for non-normally distributed variables. The level of statistical significance was set at *p* < 0.05.

## 3. Results

Patients. During the study period, 245 patients who were operated for pHPT and whose imaging investigations were US, SPS, or 4D-CT were included in the study.

Preoperative data. The median age of the patients at the time of surgical treatment was 66 years; there were 218 female (89.0%) and 27 male patients (Table 1). The preoperative biochemical characteristics were: median ionized calcium level 1.51 mmol/L; PTH 15.0 pmol/L; and phosphate 0.86 mmol/L.

**Figure 2:**
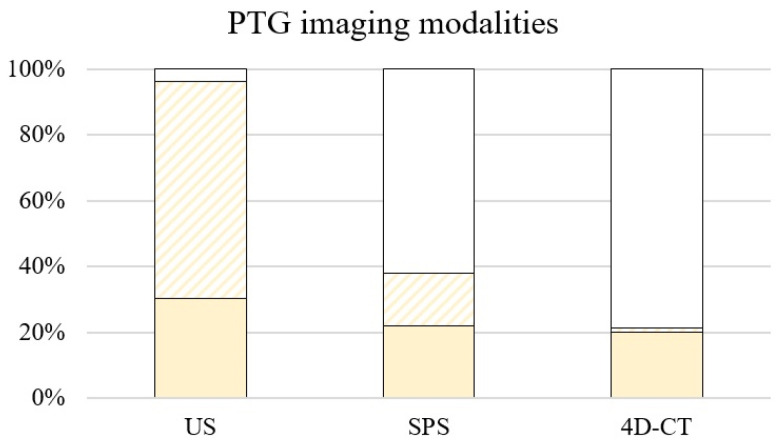
Use of PTG imaging modalities in 245 patients. Image-negative cases (striped yellow area). Successful localization of enlarged PTG (yellow area). *PTG* parathyroid gland; *US* ultrasonography; *SPS* sestamibi parathyroid scan; *4D-CT* four-dimensional computed tomography.

PTG imaging. For PTG imaging, US was carried out in 236 (96.3%, Figure 2), SPS in 93 (38.0%), and 4D-CT in 52 patients (21.2%) since its implementation in 2018. There occurred no complications associated with the use of 4D-CT imaging (e.g., development or exacerbation of renal insufficiency; intolerance of intravenous iodine based contrast media; etc.). However, for 6 patients, 4D-CT was contraindicated due to an underlying renal insufficiency or known allergy to iodine-based contrast media.

The use of 4D-CT was associated with a significantly higher rate of successful localization of enlarged PTG (49 cases, 94.2%) compared to US and SPS (74 cases, 31.4%; and 54 cases, 58.1%, respectively).

Results of surgical treatment.

According to the preoperative data gathered on PTG localization diagnostics, three groups of patients were distinguished: (1) PTG lateralization (via UH or SPS), (2) precise PTG localization (via 4D-CT), and (3) patients with non-successful localization of PTG. There were 106 patients (43.3%) in the first group, 49 in the second (20.0%), and 90 (36.7%) in the third (Table 2).

**Figure 3:**
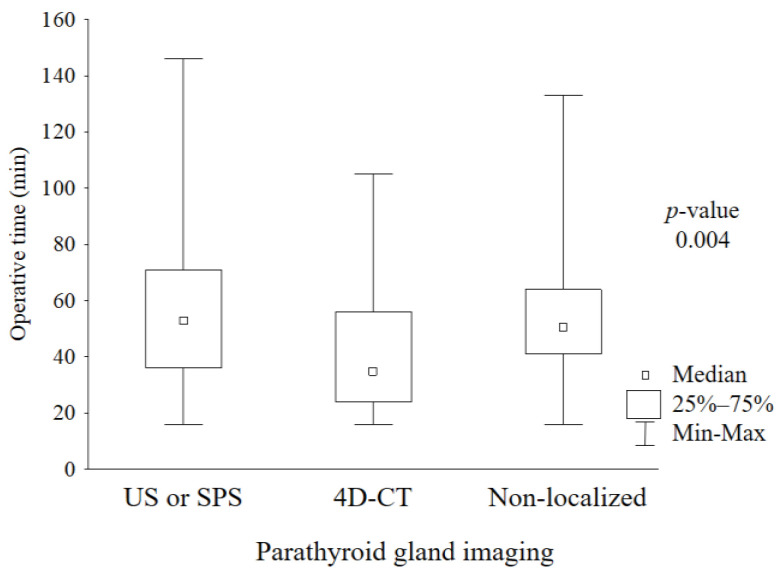
Operative time of parathyroidectomy according to preoperative localization diagnostics. *US* ultrasonography; *SPS* sestamibi parathyroid scan; *4D-CT* four-dimensional computed tomography.

More precise localizations of PTG with the use of 4D-CT led to significantly shorter median operative time, this being 35 min (Figure 3) compared to 51 min if PTG localization was not successful and 53 min after localization via US/SPS.

An assessment of the characteristics of PTX revealed a significantly higher proportion of patients in the group of non-localized PTG undergoing removal of ≥2 PTG (35.6%) compared to 23.6% and 14.3% in the US/SPS and 4D-CT groups, respectively. Although the rate of true histologically proven multiglandular disease was similar between the groups, it is obvious that in the remaining cases, ‘innocent’ PTG were removed.

The unsuccessful preoperative localization of PTG was also associated with a significantly higher rate of simultaneous thyroid resections (52.2%) compared to the US/SPS (28.3%) and 4D-CT (28.6%) groups.

Moreover, 4D-CT imaged patients showed the lowest complications rate at 2.0%, compared to 13.2% and 10.0% for the US/SPS imaged patients and the patients with failed preoperative imaging, respectively. However, the difference between the groups remained statistically non-significant (*p* = 0.076).

A short-term biochemical follow-up (one month after surgery) revealed the lowest PTH and ionized calcium levels to be in the group of 4D-CT imaged cases. Nonetheless, compared to the US/SPS imaged and non-localized cases, the difference between the groups was not significant (*p* = 0.082 for PTH, and *p* = 0.318 for ionized calcium).

Persistence of pHPT was diagnosed in three patients (6.1%) of the 4D-CT group, and in 13 (12.3%) and 7 patients (7.8%) of the US/SPS and the non-localized groups, respectively. A reoperation for persistence of hyperparathyroidism was undertaken in most cases during the first year after initial surgery. As a result of successful reoperation, a one-year biochemical follow-up demonstrated similar ionized calcium and PTH levels in all three groups.

Next, we evaluated the prevalence of complications and failure to cure as a combined metric of negative outcome suggested by Ullmann et al. [8]. There was no significant difference between the groups. Yet, the lowest rate of one or both negative events occurred in the group of 4D-CT localized PTG, this being 8.2% compared to 23.6% and 16.7% in the groups of UH/SPS localized and of non-localized patients, respectively.

**Figure 4:**
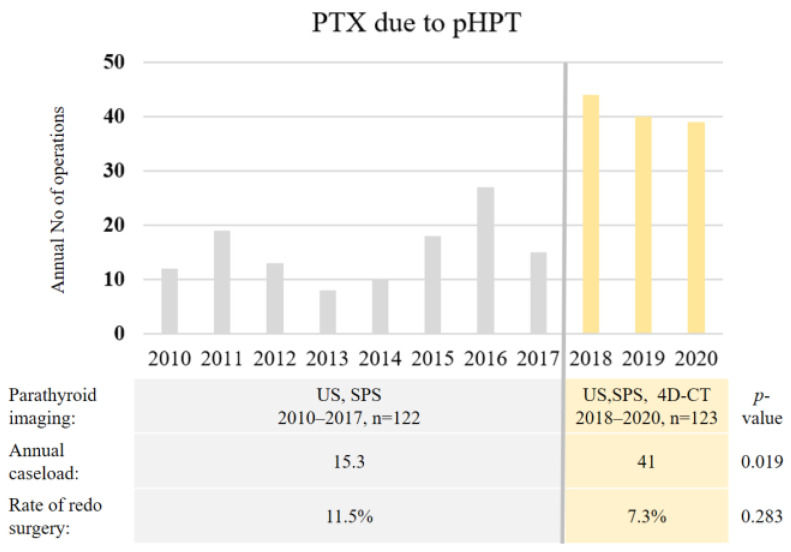
Two periods of imaging: before and after implementation of 4D-CT scan for PTG localization. Impact on the annual PTX case-load and on the rate of redo surgery. *PTX* parathyroidectomy; *pHPT* primary hyperparathyroidism; *US* ultrasonography; *SPS* sestamibi parathyroid scan; *4D-CT* four-dimensional computed tomography.

Additionally, an analysis of the results for two time frames was performed. During the first period (from 2010 to 2017), PTG was localized via US or SPS; the second period (from 2018 to 2020) started after the implementation of 4D-CT diagnostics, which was indicated for patients with inconclusive UH results. This analysis revealed two important associations with the implementation of 4D-CT (Figure 4). The need for redo surgery fell from 11.5% during the first period to 7.3% during the second period, and the annual case-load of PTX almost tripled at our institution from 15.3 cases per year for the first period to 41 cases per year for the second period.

## 4. Discussion

The present retrospective study reports an impact of the implementation of 4D-CT imaging of PTG on the results of surgical treatment of pHPT in a low-volume center.

The need for PTG imaging has been a subject of debate over decades. It has been suggested repeatedly that an experienced parathyroid surgeon is capable of localizing a vast majority of parathyroid glands without imaging assistance [17]. It has even been stated that the greatest challenge in the preoperative localization of PTG adenoma is locating an experienced parathyroid surgeon [11]. However, as parathyroid surgery also has to be performed at low or medium case-load institutions, expert surgeons are not always available [18]. Things are further complicated when we consider that this experience can only be gained from parathyroid surgery. It has been demonstrated that the surgical experience gained from non-parathyroid surgery in the same anatomical region (e.g., thyroid surgery) does not improve outcomes in parathyroid surgery. For example, centers performing a large number of thyroid but low number of parathyroid surgical procedures have a significantly higher rate of reoperative parathyroid surgery [19]. Thus, other methods need to compliment the surgeon’s experience.

Preoperativne imaging is a good way to compensate for the experience cap in lower volume centers. Frank et al. demonstrated that negative preoperative imaging correlates with reduced success rates of surgical exploration and a higher need for redo surgery [20]. An even more convincing argument for the use of extended localization diagnostics is that focused dissection in the area of enlarged PTG is associated with reduced morbidity [21]. Therefore, as the precise preoperative anatomical localization of enlarged PTG facilitates the intraoperative exploration of the culprit gland, PTG imaging has the potential to prevent a large number of BNE, as well as promote unilateral neck exploration or minimally invasive PTX instead [9].

Although the preferred sequence of imaging continues to evolve and there exists significant institutional variation, in some institutions, SPS is performed following neck US to confirm the identification of enlarged PTG, or to exclude an ectopic localization before surgical treatment is considered [22,23]. A meta-analysis assessing the value of US imaging in pHPT reported an overall pooled sensitivity of 76.1% and a positive predictive value of 93.2%; however, significant operator and center dependency was emphasized [24]. Nevertheless, despite the wide availability of SPS, several studies have reported great variation in its diagnostic accuracy, with reported sensitivity from 25.4% for US-negative patients to 78.9% for single-gland disease [9,25]. Hence, the quality of both imaging investigations and their interpretation vary dramatically between institutions, and both common localizing tests (US and SPS) may have suboptimal localizing accuracy with a considerable rate of non-localized cases [26].

In the present study, endocrinologists performed all US tests; however, the rate of successful localizations of enlarged PTG was relatively low, and the results of SPS diagnostics were only slightly better. This likely contributed to the failed preoperative imaging of enlarged PTG. Therefore, we implemented 4D-CT scan for preoperative PTG imaging in 2018 at our institution. Owing to this, we achieved a 94.2% success rate of detecting enlarged PTG. Even more importantly, there occurred several favorable changes from the clinical point of view. After the implementation of 4D-CT imaging, the need for redo surgery decreased from 11.5% to 7.3%. We also saw a move towards less extensive and more focused surgical dissection. This was illustrated by shorter PTX operative times; a lower number of patients undergoing the removal of two or more PTG; a lower rate of simultaneous thyroid surgery; and lower morbidity. However, the last difference remained statistically non-significant.

Shorter operative times for PTX in the 4D-CT imaged patients were expected, as the precise preoperative localization of enlarged PTG enabled us to use unilateral focused exploration instead of BNE in most cases. We mostly used a small (4–5 cm) central Kocher’s skin incision, followed by a unilateral dissection on the side of the enlarged PTG. The potential benefit of using Kocher’s incision is the easy conversion from unilateral dissection to classical BNE in the case of failed one-side exploration. However, according to consensus, PTX is minimally invasive when performed through an incision of less than 3 cm. Therefore, our approach has to be defined as non-minimally invasive [27,28]. Despite this, advances in preoperative imaging certainly promote the use of minimally invasive PTX, with lower complication rates and better cosmetic effect compared to BNE [29].

An additional tool facilitating a more focused dissection and better guidance of surgery was the intraoperative display of the results of 4D-CT PTG imaging. However, this statement is based on professional experience.

We believe that significantly higher rate of extirpation of two or more PTG in the group of preoperatively non-localized PTG was caused by difficulties linked to the intraoperative localization of culprit PTG, especially in cases of small adenomas. The exploration of a slightly enlarged PTG resulted in its extirpation. If the macroscopic changes were ‘not convincing’, or the frozen section did not confirm the diagnosis of PTG adenoma, further explorative dissection and extirpation of a second PTG, whether with minimal or more ‘convincing’ changes, was undertaken. As the rate of true histologically proven multiglandular disease for the groups was similar, we could conclude that the use of 4D-CT enables the reduction of the removal rate of ‘innocent PTG’.

The uncertain location of PTG probably led to the almost twofold rate of simultaneous thyroid surgery (52.2%) in patients with failed preoperative imaging of the PTG pathology, compared to cases of successfully localized enlarged PTG (28.3–28.6%). The higher rate of concurrent thyroidectomies might have been due to the removal of the protruding thyroid tissue, especially in the retrothyroideal position, which were mistaken for PTG adenomas. Ryan et al. reported a rate of 25% for simultaneous thyroid surgery, and they performed thyroid resection to optimize operative access, ensure the complete removal of involved PTG without the risk of seeding the parathyroid tissue, or more commonly, to remove synchronous thyroid pathology [30]. Thus, the optimal rate for a concurrent thyroid surgery seems to be one in every four cases of PTX. In our study we only saw similar rates for image-positive patients. However, as the rate of simultaneous thyroid surgery reported in other studies is relatively high as well (up to 50%), we believe that this is a place for further research and discussion [25].

After the implementation of 4D-CT, the observed decrease in morbidity was most probably attributable to the less extensive surgical exploration and the discarding of routine BNE. Although the difference in morbidity between the groups was obvious (2.0% for 4D-CT, 13.2% for US or SPS localized, and 10.0% for non-localized cases), it remained on the verge of statistical significance (*p* = 0.076). Nevertheless, we share the opinion that surgeons should not perform blind exploration, as positive imaging investigation can almost always be obtained to make the surgery safer [23].

There are several other diagnostic options that are suggested to improve the results of surgical treatment of pHPT. Positron emission tomography (PET) with 11C-choline has shown outstanding results with an accuracy of almost 99% [31]. However, despite its excellent accuracy, PET has considerable limitations mostly related to its cost and availability [32]. In the present study, intraoperative diagnostic tests (e.g., PTH measurement and frozen section consultation) were used relatively rarely, as they are deemed unacceptable at our institution for being too time consuming. However, we are looking forward to implementing rapid intraoperative PTH assay, as encouraged by a systematic review by Medas et al. reporting a significantly lower rate of persistent and recurrent pHPT with the use of this test [33]. This technique eliminates the need for intraoperative frozen section consultation, thereby saving time and expense [23]. In summary, we think that other novel diagnostic modalities should be used in concurrence with 4D-CT in low-volume centers.

This study had some limitations. First, the retrospective nature of this study means that there were some missing data points. Second, our cohort has quite an impressive female predominance, which can affect the generalizability of our data.

## 5. Conclusions

The use of 4D-CT imaging enabled to precisely locate almost 95% of enlarged PTG in patients with pHPT. The accurate localization and intraoperatively displayed imaging results are useful guides for surgeons to make PTX a faster and safer procedure in a low-volume center.

## Figures and Tables

**Table 1:** Preoperative characteristics of the patients.

Characteristic	Patients, n = 245
Age (y)	66 (58–74)
Female gender, n (%)	218 (89.0%)
iCa *	1.51 (1.44–1.61)
PTH ^#^	15.0 (10.4–25.3)
Phosphate ^¤^	0.86 (0.73–0.95)
Parathyroid imaging:	
− US, n (%)	236 (96.3)
− successful localization, n (%)	74 (31.4)
− SPS, n (%)	93 (38.0)
− successful localization, n (%)	54 (58.1)
− 4D-CT, n (%)	52 (21.2)
− successful localization, n (%)	49 (94.2)

Data are presented as median (with interquartile range) or percentages. Y years; iCa ionized calcium; PTH parathyroid hormone; US ultrasonography; SPS sestamibi parathyroid scan; 4D-CT four-dimensional computed tomography. * Reference value 1.16–1.32 mmol/L, ^#^ Reference value 1.6–6.9 pmol/L, ^¤^ Reference value 0.81–1.45 mmol/L.

**Table 2:** Comparison of operative and follow-up characteristics of the patients according to the results of preoperative imaging.

	Localized via US or SPS (106)	Localized via 4D-CT (49)	Non-Localized PTG (90)	*p*-Value
Operative characteristics:				
− Operative time (min)	53 (36–71)	35 (24–56)	51 (41–64)	**0.004**
− Removed ≥2 PTG, n (%)	25 (23.6)	7 (14.3)	32 (35.6)	**0.018**
− True multiglandular disease ^&^, n (%)	17 (16.0)	5 (10.2)	16 (17.8)	0.505
− PTG size (mm)	15 (10–20)	13 (10–19)	15 (8–20)	0.728
− Simultaneous thyroid surgery, n (%)	30 (28.3)	14 (28.6)	47 (52.2)	**0.001**
Complications, n (%)	14 (13.2)	1 (2.0)	9 (10.0)	0.076
− Wound hematoma, n	0	0	3	-
− RLN injury, n	3	1	1	-
− Subjective dysphonia, n	5	0	0	-
− Other	6	0	5	-
Length of stay (d)	2 (2–2)	2 (2–2)	2 (2–2)	0.272
Follow-up (30 day):				
− iCa *	1.29 (1.23–1.35)	1.25 (1.22–1.32)	1.29 (1.23–1.33)	0.318
− PTH ^#^	7.1 (5.1–12.3)	5.6 (4.3–7.6)	6.2 (3.8–11.0)	0.082
Follow-up (1 year):				
− iCa *	1.27 (1.23–1.30)	1.25 (1.20–1.32)	1.26 (1.22–1.33)	0.798
− PTH ^#^	5.4 (4.5–9.9)	5.7 (3.8–8.3)	5.4 (3.7–9.5)	0.735
Redo surgery, n (%)	13 (12.3)	3 (6.1)	7 (7.8)	0.467

Data are presented as median (with interquartile range) or percentages. US ultrasonography; SPS sestamibi parathyroid scan; 4D-CT four-dimensional computed tomography; PTG parathyroid gland; RLN recurrent laryngeal nerve; iCa ionized calcium; PTH parathyroid hormone. ^&^ Histologically proven multiglandular parathyroid gland involvement, * Reference value 1.16–1.32 mmol/L, ^#^ Reference value 1.6–6.9 pmol/L.

## Data Availability

Study data can be made available on a reasonable request for future collaborative analyses.

## Abbreviations

BNE	bilateral neck exploration
CT	computed tomography
HPT	hyperparathyroidism
iCa	ionized calcium
MRI	magnetic resonance imaging
pHPT	primary hyperparathyroidism
PET	positron emission tomography
PTG	parathyroid gland
PTH	parathyroid hormone
PTX	parathyroidectomy
RLN	recurrent laryngeal nerve
SPS	^99m^Tc sestamibi parathyroid scan
US	Ultrasonography
4D-CT	four-dimensional computed tomography
